# Duodenal Switch Is Superior to Gastric Bypass in Patients with Super Obesity when Evaluated with the Bariatric Analysis and Reporting Outcome System (BAROS)

**DOI:** 10.1007/s11695-017-2680-z

**Published:** 2017-04-24

**Authors:** Martin L. Skogar, Magnus Sundbom

**Affiliations:** Department of Surgical Sciences, Uppsala University, SE-751 85 Uppsala, Sweden

**Keywords:** Duodenal switch, Gastric bypass, Bariatric surgery, BAROS, Quality of life

## Abstract

**Background:**

It is not clear which bariatric procedure that gives the best outcome for patients with super obesity (body mass index [BMI] > 50 kg/m^2^). This study aims to compare outcomes in patients with super obesity after Roux-en-Y gastric bypass (RYGB) and duodenal switch (BPD/DS) using the Bariatric Analysis and Reporting Outcome System (BAROS) and a local questionnaire for gastrointestinal symptoms.

**Methods:**

A retrospective mail survey including 211 patients, 98 RYGB and 113 BPD/DS, with a mean follow-up time of 4 years for both groups. Gender distribution, age, and comorbidities were similar. Weight loss, changes in comorbidities, quality of life (QoL), and adverse events were registered, as well as gastrointestinal symptoms.

**Results:**

Preoperative BMI was higher in the BPD/DS group (56 ± 6.7 vs. 52 ± 4.0 kg/m^2^, *p* < 0.01); despite this, the postoperative BMI was lower (31 ± 5.5 vs. 36 ± 7.1 kg/m^2^, *p* < 0.01). The effect on diabetes was superior after BPD/DS; otherwise, both groups had a similar reduction in comorbidities. There was no difference in QoL. Adverse events were less common after RYGB (14 vs. 27%). Overall, the BPD/DS group had a superior BAROS score (4.7 ± 2.0 vs. 4.0 ± 2.1, *p* < 0.05). Dumping was more common after RYGB (*p* < 0.01), while reflux, diarrhea, fecal incontinence, and problems with malodorous flatus were more common after BPD/DS (all *p* < 0.05). Frequency of nausea/vomiting and abdominal pain were similar.

**Conclusion:**

Patients with super obesity have a better weight reduction and metabolic control with BPD/DS, at the cost of higher incidence of adverse events, compared to patients operated with RYGB.

## Introduction

The obesity-related diseases have surpassed malnourishment as global health threats today; hypertension, high fasting plasma glucose, and high body mass index (BMI) are together with smoking the top four risk factors globally according to the Global Burden of Diseases Study from 2015 [1]. Mean BMI is increasing annually [2], with the fastest growth rate seen in the highest BMI categories, and the prevalence of super obesity (BMI > 50 kg/m^2^) has become a major health issue [3]. In the USA, more than one quarter of the patients seeking bariatric surgery are super obese [4, 5].

Treating patients with super obesity is a challenge because of their medical comorbidities and technical difficulties during bariatric surgery. In addition, there is no consensus of the most appropriate procedure. Roux-en-Y gastric bypass (RYGB), the most common bariatric procedure worldwide [6], and in Sweden [7], results in massive weight loss. However, due to the high initial weight, more than half of the super obese patients are still severely obese (BMI > 35 kg/m^2^), even after a successful RYGB [8, 9]. To achieve greater weight loss in patients with super obesity, some surgeons advocate duodenal switch (BPD/DS), a procedure combining reduced intake and malabsorption of ingested nutrients. BPD/DS is also known for a superior metabolic control but requires an adequate follow-up program because of the increased risk for nutritional and gastrointestinal adverse effects [10]. Less adverse events have been reported from a center with high volume of BPD/DS [11], probably reflecting a learning curve both for the operation and the nutritional support.

Weight loss is often considered the main outcome parameter after bariatric surgery. However, definition of which amount of weight loss that should be considered a successful result varies. These lack of standards for comparison of results after bariatric surgery was pointed out as a major problem during the National Institute of Health Consensus Conference on Gastrointestinal Surgery for Severe Obesity in 1991 [12]. In addition to weight loss, the desired results after bariatric surgery are an improvement in patient’s health, both physically and mentally. Therefore, it is important to include reduction of comorbidities and improvement of quality of life (QoL), when evaluating results after bariatric surgery. For evaluation of these three domains (weight loss, comorbidities, and QoL) the Bariatric Analysis and Reporting Outcome System (BAROS) was presented by Oria et al. in 1998 [13]. The Moorhead-Ardelt Quality of Life Questionnaire (MAQ) was especially created for BAROS and correlates well with other, more widely used, QoL instruments like SF-36 [14]. A refined version of the MAQ was included in the revised BAROS in 2008 [15]. BAROS grants maximum three points in each domain (weight loss, medical condition, QoL), and occurrence of complications and reoperations deducts points. The final score classifies the results into five outcome groups, from excellent (>7–9 points) to failure (1 point or less) [13]. BAROS is considered an easy and valuable tool for evaluation of outcome after bariatric surgery, as both objective and subjective information is used [16]. It has been used for reporting results after bariatric surgery in the USA, Canada, Mexico, Brazil, and in many European countries [14].

For the patient, the need of additional surgery and rehospitalizations are of importance, as well as everyday problems, such as gastrointestinal symptoms. In combination with the weight result and quality of life, we believe that these factors determine the overall perception of the outcome.

The primary aim of this study was to compare results after RYGB and BPD/DS in patients with super obesity using BAROS. A secondary aim was to investigate possible differences of gastrointestinal symptoms after the two operations.

## Methods

All super obese patients, operated with RYGB or BPD/DS during 2003 to 2012 at a University Hospital, were identified in a local database. Eligible patients received a letter with an invitation to participate in the study and were asked to complete the BAROS quality of life questionnaire (MAQ) and our local questionnaire about additional surgery, rehospitalization, comorbidities, gastrointestinal symptoms (GI symptoms), current weight, and general perception of outcome after surgery (see Appendix). Patients were recruited during 2010 (operated 2003 to 2008), 2013 (operated 2009), and 2015 (operated 2010 to 2012) when at least 2 years had passed since their surgery. Baseline data of the patients’ preoperative BMI and comorbidities, as well as information about possible adverse events, were collected from medical records.

RYGB and BPD/DS are routinely performed at our institution in super obese patients. Type of operation performed was the patient’s choice mainly. The RYGB procedure was performed with a 100–150 cm Roux-limb and a 50 cm biliary limb. In the BPD/DS procedure, duodenum was divided distal to the pylorus and a sleeve gastrectomy was performed parallel to 36-Fr bougie. A retrocolic duodenoileostomy was constructed with a 150 cm alimentary limb and a 100 cm common limb. The remaining small bowel formed the biliary limb.

Weight change was calculated as the percentage of excess body mass index loss (%excess BMI loss) using the formula (preoperative BMI − follow-up BMI) × 100/(preoperative BMI − 25). Weight gain above initial weight deducted one point and %excess BMI loss of 0 and 24% scored no points. If %excess BMI loss was between 25 and 49%, one point was scored; between 50 and 74%, two points; and finally, 75 to 100% scored three points. Comorbidities analyzed were diabetes, hypertension, cardiovascular disease, sleep apnea, and dyslipidemia. The presence of a comorbid disease was determined by the use of medication, and remission was defined as cessation of all medications for the specific disease (CPAP for sleep apnea). Changes were scored accordingly: aggravation (minus one), unchanged (no points), improved (one point), one major comorbidity resolved and others improved (two points), and all major comorbidities resolved (three points). A translated version of MAQ was used where each of the six questions were scored from −0.5 to +0.5 giving a total score between −3 and +3 points. All complications were analyzed from medical records. Major complications, defined in BAROS as reoperation or a complication with prolonged hospital stay/rehospitalization ≥7 days, deducted one point from the final score, while minor complications, i.e., complication with prolonged hospital stay/rehospitalization <7 days, deducted 0.2 points. A BAROS score was calculated for each patient, which classified the result as failure (<1 point), fair (>1 to 3 points), good (>3 to 5 points), very good (>5 to 7 points), or excellent (>7 to 9 points). In patients not suffering from any obesity-related comorbidity preoperatively, the modified scoring key, excluding changes in comorbidities (failure ≤ 0, fair > 0–1.5, good > 1.5–3, very good > 3–4.5, and excellent > 4.5–6) [13], was used.

### Statistics

Values are presented as mean ± standard deviation (SD) unless otherwise stated. A *p* value of <0.05 was considered statistically significant. For comparison between groups, Mann-Whitney test was used for nonparametric data and unpaired *t*-test for parametric data. Chi-squared test, or when applicable, Fischer’s exact test was used for comparison of gender distribution, comorbidities, and complication rates. GraphPad Prism version 6.0f (GraphPad Software, La Jolla, CA, USA) was used for the statistical analysis.

## Results

In total, 333 eligible patients were identified whereof 212 patients returned the questionnaires. After exclusion of one patient with incomplete response form, the final analysis comprised of 211 patients (98 RYGB and 113 BPD/DS), a total response rate of 63%. RYGB was performed as laparoscopic surgery in 11 patients; otherwise, all procedures were open surgery. Mean follow-up time was 4 years for both groups (4.0 ± 1.1, range 2.2 to 6.5 years for RYGB, vs. 4.0 ± 1.0 years, range 2.1 to 7.3 for BPD/DS). Groups were similar concerning gender distribution, age, and comorbidities, but preoperative BMI was lower in the RYGB group (Table 1).

**Table 1:** Characteristics, changes in body mass index (BMI) and comorbidities

	Preoperative			Change			Postoperative		
	RYGB (*n* = 98)	BPD/DS (*n* = 113)	*P* value ^a^	RYGB	BPD/DS	*P* value^b^	RYGB (*n* = 98)	BPD/DS (*n* = 113)	*P* value^c^
Female no. (%)	68 (69)	66 (58)	0.12	–	–	–	–	–	–
Age (years)	40 ± 10	40 ± 9.9	0.96	–	–	–	–	–	–
BMI (kg/m^2^)	52 ± 4.0	56 ± 6.7	<0.01	−62%^d^	−79%^d^	<0.01	36 ± 7.1	31 ± 5.5	<0.01
Any comorbidity (%)	61 (62)	66 (58)	0.58	−32 (−52)	−36 (−55)	0.45	29 (30)	30 (27)	0.65
Diabetes no. (%)	14 (15)	27 (25)	0.08	−10 (−71)	−25 (−93)	<0.05	4 (4)	2 (2)	0.42
Hypertension no. (%)	35 (38)	40 (37)	1.00	−13 (−37)	−13 (−33)	0.88	22 (24)	27 (25)	0.87
Cardiovascular disease no. (%)	3 (3)	1 (1)	0.34	+2 (+67)	+1 (+100)	0.60	5 (5)	2 (2)	0.25
Sleep apnea no. (%)	18 (20)	25 (23)	0.61	−15 (−83)	−19 (−76)	0.85	3 (3)	6 (6)	0.51
Dyslipidemia no. (%)	14 (15)	18 (17)	0.85	−7 (−50)	−14 (−78)	0.25	7 (8)	4 (4)	0.35

*RYGB* Roux-en-Y gastric bypass, *BPD/DS* duodenal switch

^a^Between groups, preoperative

^b^Between groups, difference in change

^c^Between groups, postoperative

^d^% Excess BMI loss

An analysis of 112 of the 121 nonresponders was possible from local data entered into the Scandinavian Obesity Surgery Registry (SOReg). The nonresponding RYGB group (*n* = 55) consisted of more men (53 vs. 31%, *p* < 0.01), but age, preoperative BMI, and comorbidities did not differ compared to the responding RYGB group. The nonresponding BPD/DS group (*n* = 57) was somewhat younger (36 years, *p* < 0.05), but gender distribution and BMI were similar to the responding BPD/DS group. Prevalence of diabetes was lower in the nonresponding BPD/DS group (7%, *p* < 0.01); all other comorbidities were similar to the responding group.

### Weight Loss

The %excess BMI loss was 62 ± 23% for the RYGB group, while the BPD/DS group lost 79 ± 17% of their excessive BMI, which resulted in a significant lower postoperative BMI in the BPD/DS group (31 vs. 36 kg/m^2^, *p* < 0.01). There were 48 patients (49%) in the RYGB group and 27 patients (24%) in the BPD/DS group who were severely obese (BMI > 35 kg/m^2^) even after weight loss.

### Improvement of Medical Conditions

In 61 patients (62%) in the RYGB group and in 66 patients (58%) in the BPD/DS group, obesity-related comorbidities were noted in the medical records preoperatively. Both groups had a significant reduction in diabetes and sleep apnea (all *p* < 0.05). The BPD/DS group also had a significant reduction in dyslipidemia (*p* < 0.01), while the reduction in dyslipidemia after RYGB and the reduction in hypertension for both groups failed to reach statistical significance. The effect on diabetes was superior after BPD/DS, while changes in the other comorbidities were similar between groups. No positive change of cardiovascular disease was seen; another two patients in the RYGB group and one patient in the BPD/DS group had congestive heart failure at follow-up. No differences in comorbidities were seen between groups postoperatively (Table 1).

### Complications

Fourteen patients (14%) had one or more complications in the RYGB group and 31 patients (27%) in the BPD/DS group. One patient in the RYGB group had a perforated ulcer, and some months later, she also had an internal herniation, thus two major complications with reoperation. In the BPD/DS group, three patients had more than one complication. One patient was reoperated early because of a significant abdominal bleeding and a concomitant biliary leakage from the divided duodenal stump, thus two major complications. One patient with an abdominal abscess developed an incisional hernia, and later a peptic ulcer. The third patient suffered from severe abdominal pain after a reoperation for bowel obstruction. The overall complication rate was lower after RYGB (8 minor and 7 major vs. 18 minor and 17 major, *p* < 0.05). All complications are listed in Table 2.

**Table 2:** Complications after surgery

	RYGB		BPD/DS	
Complication	Minor	Major	Minor	Major
Abdominal abscess	–	–	1	–
Abdominal pain	3	–	3	1
Atrial fibrillation	–	–	1	–
Biliary leakage	–	–	–	2
Bleeding	3	–	2	2
Bowel obstruction	–	2	–	6
Dehydration	–	–	1	–
DVT/PE	–	1	–	–
Incisional hernia	–	3	–	5
Malnutrition	1	–	5	1
Peptic ulcer	1	1	4	–
Seroma	–	–	1	–
Total	8	7	18	17

Minor = complication with prolonged/rehospitalization of ≤7 days. Major = complication with prolonged/rehospitalization ≥7 days or reoperation

*RYGB* Roux-en-Y gastric bypass, *BPD/DS* duodenal switch, *DVT/PE* deep venous thrombosis/pulmonary embolism

### Quality of Life and BAROS Score

In the first category (weight loss), the RYGB group scored lower than the BPD/DS group, while scores were similar in the second category (comorbidities). If patients without comorbidities (38% of RYGB and 42% of BPD/DS) were excluded, the average score was 1.6 for RYGB and 1.9 for BPD/DS (*p* = 0.15). The third category (QoL) was also similar between the groups. A subgroup analysis of patients who had suffered an adverse event showed that these patients’ score on the MAQ did not differ significantly compared to those without an adverse event (RYGB group 0.9 vs. 1.2, *p* = 0.52, and BPD/DS group 1.0 vs. 1.3, *p* = 0.47). The RYGB group had less score deduction for complications (*p* < 0.05). Overall, the BPD/DS group had a higher BAROS score (Table 3).

**Table 3:** BAROS score

	RYGB (*n* = 98)	BPD/DS (*n* = 113)	*P* value
Weight loss	1.9 ± 0.8	2.6 ± 0.6	<0.01
Change in comorbidities	1.0 ± 1.2	1.1 ± 1.2	0.67
Quality of life	1.1 ± 1.1	1.2 ± 1.2	0.51
Complications	−0.1 ± 0.2	−0.2 ± 0.4	<0.05
Total	4.0 ± 2.1	4.7 ± 2.0	<0.05

*RYGB* Roux-en-Y gastric bypass, *BPD/DS* duodenal switch

**Fig. 1:**
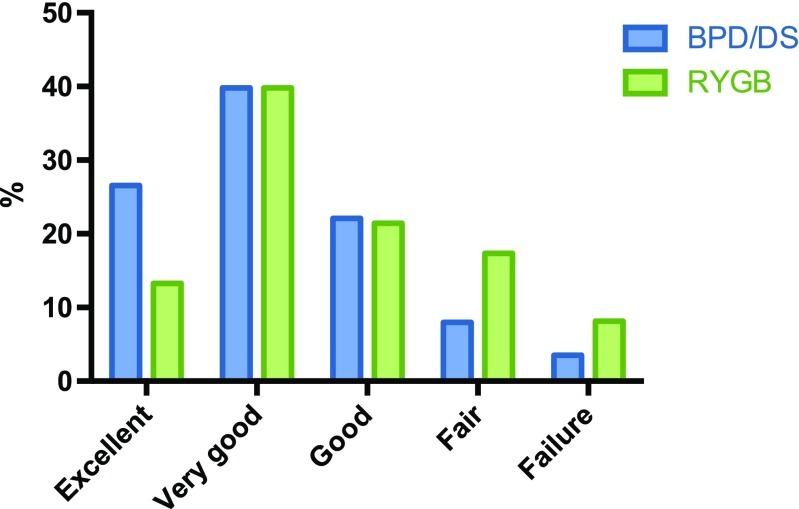
The percentage distribution of patients in the five categories. *RYGB* Roux-en-Y gastric bypass, *BPD/DS* duodenal switch

When categorizing the postoperative result into one of the five outcome categories, there were more patients in the BPD/DS group with “excellent” (27 vs. 13%) as outcome, and fewer patients categorized as “failure” (4 vs. 8%) or “fair” (8 vs. 17%) compared to the RYGB group (Fig. 1).

### Gastrointestinal Symptoms

**Fig. 2:**
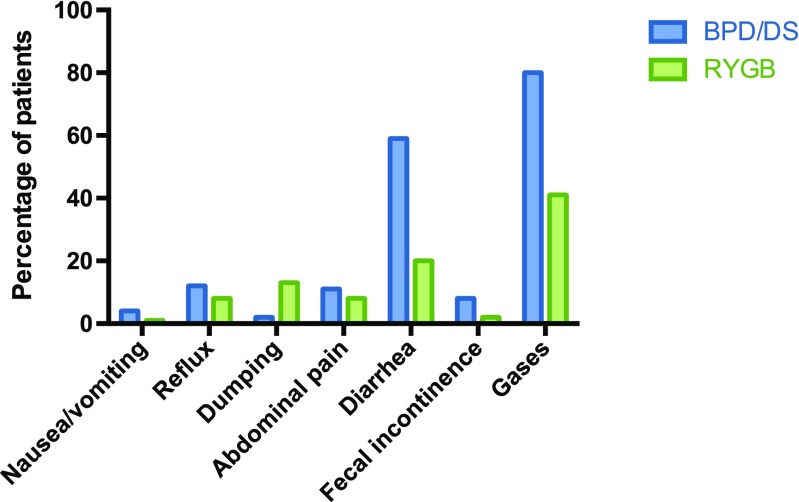
Percentage of patients with GI symptoms weekly or more often. *RYGB* Roux-en-Y gastric bypass, *BPD/DS* duodenal switch

Symptoms of rapid gastric emptying (dumping) were more common after RYGB (*p* < 0.01), 13% had symptoms weekly or more often. Gastroesophageal reflux, diarrhea, fecal incontinence, and problems with malodorous flatus were more common after BPD/DS (all *p* < 0.05). The most prominent GI symptoms after BPD/DS were diarrhea and malodorous flatus (59 and 80% daily or weekly, vs. 20 and 41% after RYGB). Frequency of nausea/vomiting and abdominal pain was similar (Fig. 2).

### Overall Perception of Outcome After Surgery

On the question where patients were asked to rate their overall perception of the outcome after surgery, 58% in the RYGB group and 62% in the BPD/DS group said they were “satisfied” or “very satisfied.” In both groups, 90% of the patients would recommend bariatric surgery to other patients suffering from severe obesity.

## Discussion

In this study, where BAROS score was analyzed in 211 super obese patients, the postoperative outcome was more favorable after BPD/DS compared to RYGB. BPD/DS was characterized by superior weight loss and better effect on diabetes, but at the cost of more gastrointestinal symptoms.

The main reason for better BAROS score after BPD/DS was a significant better result on weight loss compared to RYGB. Almost half (49%) of the patients in the RYGB group were still severely obese (BMI > 35) at follow-up. The observed weight loss after RYGB and BPD/DS in this study was not surprising since several previous studies comparing the two methods in patients with super obesity have found similar results [10, 17, 18], and it has previously been pointed out that weight loss is more sustained after BPD/DS [19]. In a recent American study by Strain et al. [20], a sustained BMI reduction from 53.4 to 31.5 kg/m^2^ was observed throughout the 9 years of observation after BPD/DS. Two previous studies [8, 9] observed that more than 50% of the super obese patients still had a BMI >35 kg/m^2^ after RYGB. Concerning this poor weight loss after RYGB, we believe that it is probably easier to revise a few percent of BPD/DS patients (1 of 113 in this study) suffering from malnutrition, than to reoperate 50% of the patients after RYGB with poor weight loss.

The effect on comorbidities was impressive for both groups, with a superior effect on diabetes after BPD/DS. Previous studies of super obese patients have found higher remission rates both of diabetes, dyslipidemia, and hypertension after BPD/DS compared to RYGB [18, 21], but this study could only confirm a better effect on diabetes after BPD/DS. A Norwegian randomized clinical trial (RCT) between RYGB and BPD/DS in patients with BMI 50 to 60 kg/m^2^ (*n* = 31 and 29, respectively) observed significantly lower hemoglobin A_1C_ and serum triglycerides after BPD/DS, but complete remission of diabetes and metabolic syndrome were similar between groups [10]. In the study by Topart et al. from 2013 [19], they found similar remission rates of diabetes and sleep apnea, but higher remission rate of hypertension after BPD/DS (*n* = 56) compared to RYGB (*n* = 65) in super obese patients 3 years after surgery. In the study by Suter et al. [8], they found a similar reduction of comorbidities comparing results after RYGB in patients with morbid obesity (BMI 34–49.9 kg/m^2^) to patients with super obesity (BMI 50–73 kg/m^2^) despite less satisfactory weight loss in the super obese patients. Remission rates of abnormal glucose metabolism, hypertension, and sleep apnea were remarkably high in their study, >90% for both groups. A reason might be the preoperative education course and intense follow-up by a dedicated team in combination with longer Roux-limb (150 cm) in patients with BMI >48 kg/m^2^. They conclude that a high weight loss per se is not that important for resolution of comorbidities. This statement is supported by the findings of Prachand et al. [21], where they observed a weight-loss-independent improvement of comorbidities after RYGB and BPD/DS, but they also found a significant higher remission of diabetes, dyslipidemia, and hypertension after BPD/DS compared to RYGB, as mentioned above.

On the other hand, the BPD/DS group suffered more adverse events and had more GI symptoms, especially diarrhea and malodorous flatus. These results are in line with the previously mentioned RCT [10] and a systematic review and meta-analysis of RYGB and BPD/DS by our group [22]. In contrast, Dorman et al. [18] concluded that complication and adverse event rates were similar between RYGB and BPD/DS, based on their findings of different panorama of complications between groups.

The QoL (MAQ score) did not differ between groups in the present study, despite more complications and GI symptoms after BPD/DS. Perhaps the superior weight result is more important to the patients. The idea is supported by the findings of Batisis et al. [23], who found that percent of weight loss is highly associated with higher quality of life. In the previously mentioned American study [20], a significant improvement in QoL was observed 1 year after surgery and it was well maintained for the 9 years of observation, with SF-36 scores similar to community norms after BPD/DS. MAQ scores observed in this study (1.1 and 1.2) were lower than those observed in a comparative study of patients with BMI <60 kg/m^2^ to patients with BMI >60 kg/m^2^ (1.8 and 1.7, respectively), after laparoscopic RYGB (LRYGB) [24]. However, BAROS survey response was only obtained in 35% of the patients in that study, making it difficult to draw any firm conclusions, and the laparoscopic approach could perhaps also explain the difference. Our patients were operated from 2003 to 2012, which, together with their high BMI, explains why only 11 patients were operated laparoscopically. During that time period and onwards, laparoscopy has become the most common approach in Sweden (97% of all RYGB in 2014 (7)). MAQ scores observed in this study are very similar to those observed in a study from 2004 of more than 800 BPD/DS patients 15 years after surgery, even though they used the older version of MAQ [25].

To the best of our knowledge, this is the first study comparing outcomes after RYGB and BPD/DS in super obese patients, using BAROS. Other studies of super obese patients have observed higher total BAROS score for RYGB than we found in this study [24, 26, 27]. However, those studies were of LRYGB and proportions of comorbidities were somewhat different to our RYGB group. One has to remember that 38% of the patients in the RYGB group and 42% of the patients in the BPD/DS group did not suffer from any preoperative comorbidity and therefore scored according to the modified scoring key were the maximum score is 6 [13]. When looking at the final scoring category instead, our RYGB patients did better than those in the study by Farkas et al. [24], were none of the patients were categorized as having an “excellent” or “very good” outcome. The outcome of our BPD/DS group (27% “excellent,” 40% “very good,” and 22% “good”) are in line with a study of Guedea et al. [28], where they studied a similar group of patients (*n* = 74, mean BMI of 54 kg/m^2^) 5 years after BPD/DS and found “excellent” outcome in 24%, “very good” in 42%, and “good” in 31%. Our BPD/DS group also scored very similar to the patients in the previously mentioned 15-year follow-up after BPD/DS [25]. Compared to other bariatric procedures, our RYGB and BPD/DS patients scored higher than patients after adjustable gastric banding in a 4- and 7-year follow-up (*n* = 404, BMI 42.1 ± 0.2 kg/m^2^) [29, 30], but lower than patients after laparoscopic sleeve gastrectomy (LSG) (*n* = 23, BMI 40.7 ± 6.6 kg/m^2^) [31]. However, results are hard to compare since none of the studies were of super obese patients and all patients in the study of LSG had diabetes preoperatively. In a study of 5-year results after LSG [32], with patient more similar to ours (mean BMI = 50.7 kg/m^2^), they found lower BAROS score than we did, mainly depending on less satisfactory weight loss. In a recent study by Janik et al. [33], they observed similar results of QoL (using MAQ) between LRYGB and LSG and both operated groups scored higher than a nonoperated control group.

This study has some limitations that need consideration. First, this is a retrospective, nonrandomized study with a response rate of 63%. However, the sensitivity analysis comparing responders and nonresponders demonstrated fewer men in the responding RYGB group and older patients in the responding BPD/DS group, two differences unlikely to overestimate the treatment effect. Second, groups were different concerning preoperative BMI, but despite higher initial BMI, BPD/DS resulted in a lower postoperative BMI compared to RYGB, thus demonstrating the superior effect on weight loss. Third, the presences of comorbid diseases were determined by the use of specific drugs, with no attention to blood samples for glycemic control or blood lipids. This definition contributes to an uncertainty in the analysis of changes in comorbidities. In addition, we have not analyzed the remaining obesity-related comorbidities included in the BAROS (osteoarthritis, infertility, urinary stress incontinence, and depression) because they were uncertain variables to analyze from the medical records. Fourth, in this study, all but 11 patients were operated with an open approach for reasons previously mentioned. The open approach is associated with higher incidence of incisional hernia [34] (eight in the present study) compared to the laparoscopic approach, which is standard today.

### Conclusion

Patients with super obesity have superior weight reduction and a better effect on diabetes with BPD/DS. This occurs at the cost of more adverse events and GI symptoms, but with similar QoL, compared to patients operated with RYGB. We therefore believe that the choice of bariatric procedure must be made in close agreement between the surgeon and a well-informed patient.
